# Impact of Noncoaxial Transcatheter Aortic Valve Implantation on Clinical Outcomes

**DOI:** 10.1016/j.jacadv.2025.101834

**Published:** 2025-05-22

**Authors:** Domenico Angellotti, Marc Pfluger, Annette Maznyczka, Daijiro Tomii, Masaaki Nakase, Stefan Stortecky, Jonas Lanz, Daryoush Samim, David Reineke, Fabien Praz, Stephan Windecker, Thomas Pilgrim

**Affiliations:** a Department of Cardiology, Bern University Hospital, Bern, Switzerland; b Department of Advanced Biomedical Sciences, University of Naples Federico II, Naples, Italy; c Department of Cardiovascular Surgery, Bern University Hospital, Inselspital, University of Bern, Bern, Switzerland

**Keywords:** axial angle, bioprosthetic valve failure, coaxial implantation, early safety, transcatheter aortic valve replacement

## Abstract

**Background:**

Noncoaxial placement of transcatheter heart valves (THVs) relative to the native aortic annulus is occasionally detected on fluoroscopy after transcatheter aortic valve replacement (TAVR).

**Objectives:**

This study aimed to evaluate the impact of noncoaxial TAVR deployment on clinical outcomes.

**Methods:**

We retrospectively evaluated consecutive patients undergoing transfemoral TAVR in the Bern transcatheter aortic valve implantation registry. Coaxiality between the native annulus and the THV was measured using the 3-cusp view on fluoroscopic images and was defined as the angle between a line intersecting the lower points of native cusps and a line intersecting the lower hinge points of the prosthesis frame. Patients were categorized according to tertiles of coaxiality.

**Results:**

Among 2,025 patients (mean age 81.6 ± 6.5 years, mean Society of Thoracic Surgeons Predicted Risk of Mortality 4.2% ± 3.3%) undergoing TAVR with contemporary devices between February 2014 and June 2023, the mean axial angulation of the device relative to the native annulus was 4.1°. According to Valve Academic Research Consortium-3 criteria, patients in the highest tertile of THV axial angle (range, 4.8-21.7°) had reduced device early safety (56.2% vs 82.7%; aHR: 0.67; 95% CI: 0.59-0.76; *P* < 0.001) and higher rates of stage-2 bioprosthetic valve failure at 1-year follow-up (1.5% vs 0.7%; HR: 3.47; 95% CI: 1.26-9.54; *P* = 0.016), compared to those with coaxial valve positioning (range, 0.1-3.0). Left ventricle outflow tract calcium volume, predilatation, and valve type were independent predictors of noncoaxial valve implantation.

**Conclusions:**

Noncoaxial THV deployment is associated with impaired valve early safety and increased risk of bioprosthetic valve failure 1 year after TAVR.

Paravalvular regurgitation (PVR) and atrioventricular conduction disturbances requiring the implantation of a permanent pacemaker (PPM) remain among the most common complications after transcatheter aortic valve replacement (TAVR) and have been associated with an increased risk of death.^1^, ^2^, ^3^, ^4^, ^5^, ^6^, ^7^ Both complications can be mitigated by precise device positioning and stable valve deployment.^8^ Transcatheter aortic valve positioning has been facilitated by technological refinement of delivery catheters and devices, as well as improved implantation techniques.^9^^,^^10^ The behavior of the prosthesis within the aortic annulus is a complex interplay of several factors, including the anatomy of the aortic annulus and the left ventricular outflow tract (LVOT), the degree and distribution of calcification of the aortic valve leaflets, and the ability of the nitinol stent to exert its radial force.^11^^,^^12^ Coaxial implantation of the bioprosthesis warrants an even distribution of shear stress to the leaflets. Suboptimal transcatheter heart valve (THV) positioning, including noncoaxial valve implantation relative to the native annulus, can be observed on fluoroscopy after TAVR deployment and may be a marker of suboptimal valve performance. There are, however, no dedicated studies demonstrating an association between reduced coaxiality between prosthesis and native annulus and adverse clinical outcome. The present study aimed to evaluate the effect of noncoaxial THV implantation on clinical outcomes in patients undergoing transfemoral TAVR with contemporary devices.

## Methods

### Population

**Figure 1:**
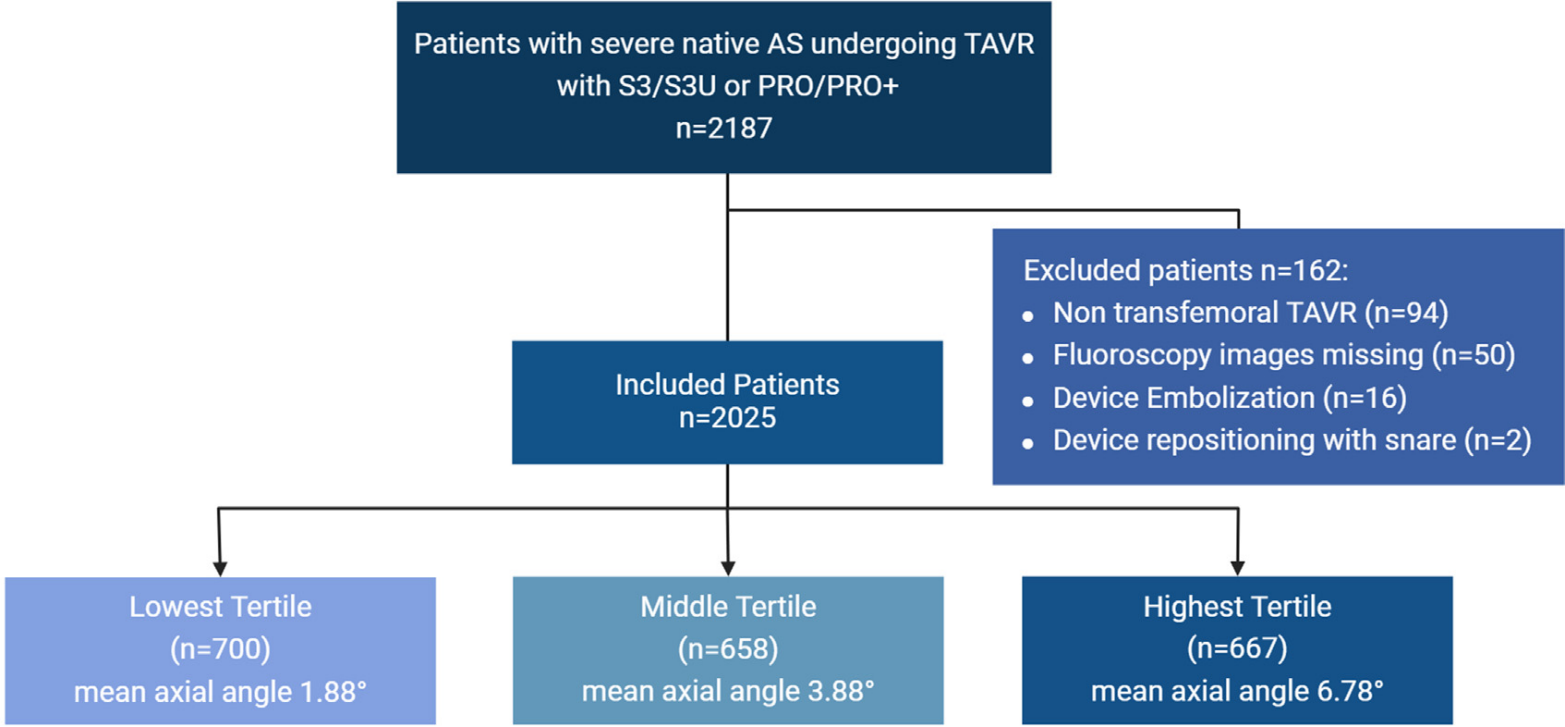
Study Flowchart Flow diagram illustrating the number of patients included and their distribution across tertiles of axial angle. AS = aortic stenosis; TAVR = transcatheter aortic valve replacement.

Bern transcatheter aortic valve implantation (TAVI) is a prospective registry that includes consecutive patients undergoing TAVR at Bern University Hospital. It is part of the nationwide Swiss TAVI Registry (NCT01368250). The registry was approved by the ethics committee, and all participants provided written informed consent for participation. For the purpose of the present analysis, we included patients with severe native aortic stenosis (AS) undergoing transfemoral TAVR with contemporary devices that had an adequate fluoroscopic 3-cusp view for analysis of the angulation between the bioprosthesis relative to the native aortic annulus. Patients with device embolization requiring the implantation of a second valve or conversion to surgery, as well as patients with inadequate fluoroscopic recordings post-TAVR were excluded (Figure 1).

### Data collection

All baseline clinical, procedural, and follow-up data were prospectively recorded in a web-based database maintained by the Clinical Trials Unit at the University of Bern. Standardized baseline echocardiographic and computed tomographic (CT) imaging data were independently re-evaluated by specialized imaging experts and incorporated into the database. The device landing zone calcium volume was quantified in the contrast images using a predefined Hounsfield unit threshold of 850, as previously validated.^13^ Aortic valve disease was assessed using transthoracic echocardiography conducted by experienced echocardiographers. The severity of AS was evaluated based on peak velocity, mean gradient, and the calculation of the aortic valve area using the continuity equation, following current guidelines.^14^^,^^15^ Echocardiographic follow-up is recommended at discharge and at 30-day and at 1-, 5-, 10- and 15-year post-TAVR. Additional/unplanned echocardiography is performed when clinically indicated. Echocardiographic data from planned and unplanned echocardiography are prospectively recorded in a web-based database by local teams using a standardized case report form. Clinical follow-up data at 30 days, 1 year, and the longest available follow-up post-TAVR were collected through standardized interviews, documentation from referring physicians, and hospital discharge summaries. THV sizing was retrospectively evaluated based on the implanted THV size and CT-derived aortic annulus area or perimeter.^16^ According to the manufacturers' instructions, oversizing and undersizing were defined as valve size exceeding >5% the upper and lower border of the recommended range, respectively. TAVR endpoints and adverse events were assessed according to Valve Academic Research Consortium-3 (VARC-3) definitions.^17^ Adverse clinical events were blinded for patient details and adjudicated following review of original source documents by a dedicated clinical event committee. An independent Clinical Trials Unit oversaw central data monitoring to ensure data completeness and accuracy.

### Assessment of prosthetic valve coaxiality relative to the native aortic annulus

**Figure 2:**
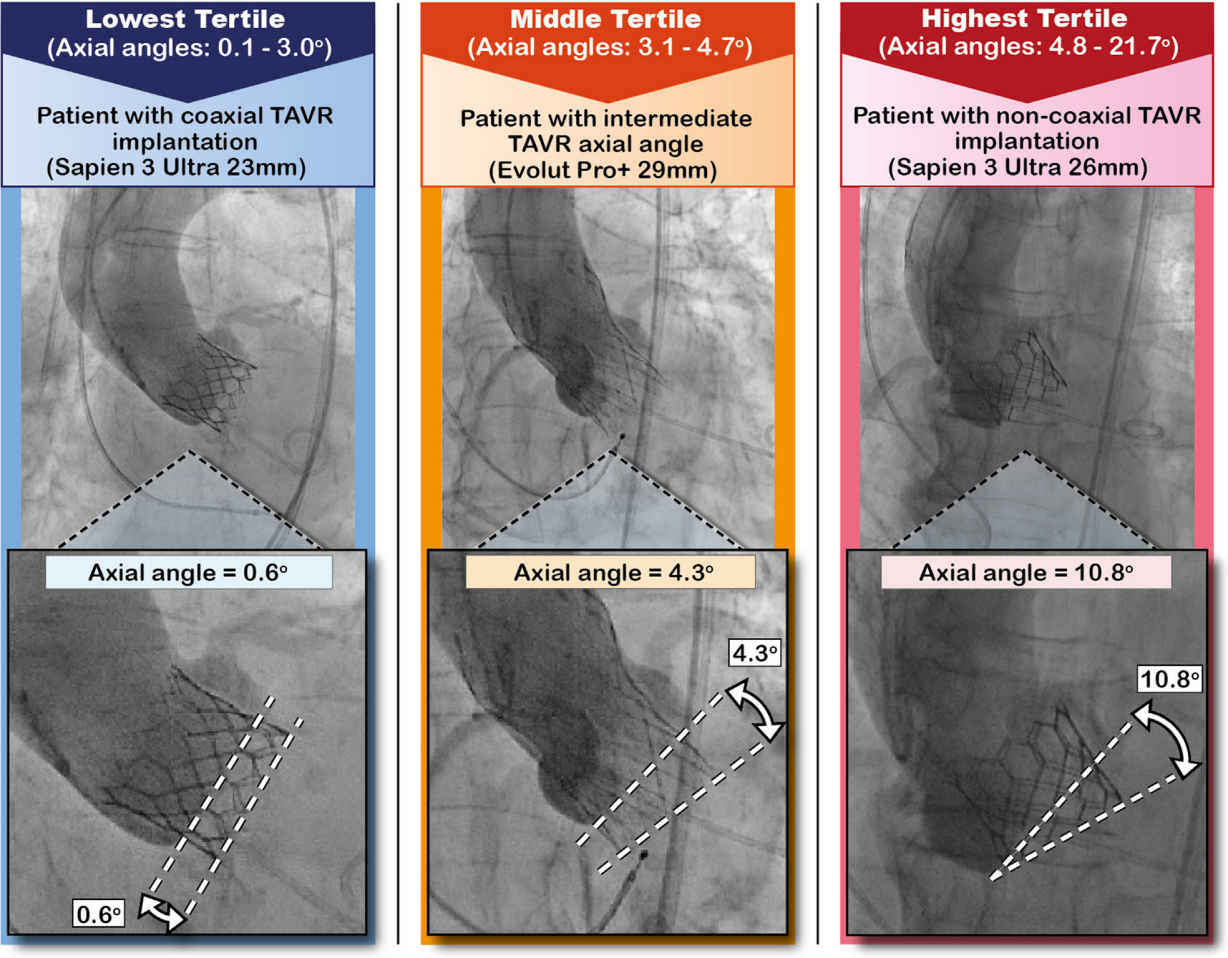
Representative Case Examples Three patients who underwent TAVR for severe native aortic stenosis. The patient on the left panel had a transfemoral Sapien 3 Ultra 23 mm valve and implantation of the prosthetic valve was coaxial (axial angle 0.6°). The patient in the middle panel had an Evolut Pro+ 29 mm valve and the prosthetic valve axial angle was intermediate (4.3°). The patient on the right panel had a Sapien 3 Ultra 26 mm valve and implantation of the prosthetic valve was noncoaxial (axial angle 10.8°). Abbreviation as in Figure 1.

Coaxiality was assessed on two-dimensional fluoroscopy. At the TAVR procedure, noncontrast cine images in the 3-cusp view were obtained immediately after THV deployment. A single still frame of the aortic angiogram taken after THV implantation was analyzed for each patient by 2 investigators (D.A. and M.P.). The image with best quality, that is, the one with the least motion artifacts, was identified. The axial angle was defined as the angle between 2 lines: 1) a line drawn through the lowest points of the native cusps, representing the orientation of the native valve annulus; and 2) a line intersecting the lower hinge point of the inflow portion of the prosthesis frame, defining the orientation of the prosthetic valve (Figure 2). The axial angle quantifies the difference in orientation between the native annulus plane and the prosthetic valve frame. This angle was measured using the 3-cusp view projection for consistency. Angiograms were reviewed before any statistical analysis, with fluoroscopy readers blinded to clinical data and outcomes. For a subset of 200 randomly selected patients, interobserver variability for the axial angle was found to be satisfactory (interobserver intraclass correlation coefficient: 0.88; 95% CI: 0.87-0.91; *P* < 0.001).

### Outcomes of interest

The outcome of interest was VARC-3 device early safety 30 days post-TAVR. Secondary outcomes included stage-2 bioprosthetic valve failure (BVF) at 1-year follow-up after TAVR. According to VARC-3 criteria, early safety at 30 days was defined as freedom from: 1) all-cause mortality; 2) stroke; 3) VARC type 2 to 4 bleeding; 4) major vascular, access-related, or cardiac structural complication; 5) acute kidney injury stage 3 or 4; 6) moderate or severe PVR; 7) new PPM implantation; and 8) valve reintervention. Stage-2 BVF was defined as transcatheter or surgical reintervention related to the device. Secondary analysis also included the incidence of all-cause mortality, cardiovascular mortality, cerebrovascular events, and hemodynamic valve deterioration at both 1 year and longest follow-up available.

### Statistical analysis

Normality was assessed using the Shapiro-Wilk test, skewness, and kurtosis indices. Qualitative variables are reported as counts and percentages and compared using the chi-square test for independent variables. In the case of normal distribution, quantitative values are expressed as mean ± SD and compared using Student's *t*-test or analysis of variance; otherwise, values appear as median (IQR) and were compared with the Mann-Whitney or Kruskal-Wallis test, as applicable. Linear regression analysis, informed by clinical rationale, was conducted to examine the association between the variables and axial angle. Results of the binary logistic regression analyses are presented as OR and adjusted OR (aOR), with 95% CIs. The linearity assumption between continuous independent variables and the log-odds of the outcome was assessed using cubic splines. Cox proportional hazard regression analysis was performed to assess HR for time-dependent outcomes across tertiles of coaxiality. Such impact was evaluated employing univariable analysis and multivariable analysis adjusting outcomes for device type, age, sex, and Society of Thoracic Surgeons Predicted Risk of Mortality score (aHR). For each specific outcome, a cause-specific Cox model was used, considering death as a censoring event. The proportional hazard assumption was verified using Schoenfeld residuals tests. Adjudicated events are expressed as counts and incidence rates. All-cause death is computed using Kaplan-Meier method and compared using the log-rank test. Gray's test was used to construct and compare the cumulative incidence considering all-cause death as a competing risk for stroke, stage-2 BVF and cardiovascular death at 1 year. Interobserver variability was evaluated with an interclass correlation coefficient (2-way mixed-effects model, random targets, fixed raters). A *P* value <0.05 was considered to indicate statistical significance. Analyses were performed with R studio, version 4.1.2 (R Core Team) and IBM SPSS Statistics, version 26 (IBM Corporation).

## Results

Among 2,187 consecutive patients with severe AS who underwent TAVR with contemporary devices between February 4, 2014, and June 30, 2023, 162 patients were excluded because of alternative access (n = 94), inappropriate fluoroscopic images (n = 50), or device embolization or migration (n = 18). Of the remaining 2,025 patients, 1,566 (77.3%) received a balloon-expandable valve (Sapien 3/Ultra) and 459 (22.7%) received a self-expanding valve (Evolut PRO/PRO Plus). Patients were categorized into tertiles based on the angle of the prosthetic valve relative to the native aortic annulus: 700 patients were in the lowest tertile (axial angle range, 0.1-3.0°, mean 1.9°) and were considered to have coaxial valve deployment; 658 patients were in the intermediate tertile (range, 3.1-4.7°, mean 3.9°), and 667 in the highest tertile (range, 4.8-21.7°, mean 6.8°). Patients in the highest tertile were considered to have noncoaxial valve implantation.

### Clinical characteristics

The clinical characteristics of the study cohort are summarized in Table 1. The mean age of the population was 81.6 ± 6.5 years, 43.0% of the patients were female, and the mean Society of Thoracic Surgeons Predicted Risk of Mortality score was 4.2% ± 3.3%. Women were significantly more represented in the highest tertile, that is, patients with noncoaxial valve deployment, compared to those in the intermediate and those in the lowest tertiles, that is, patients with coaxial valve implantation (46.8% vs 42.9% vs 39.6%, respectively, *P* = 0.027). Patients in the highest tertile also had higher LVOT calcium volume on pre-TAVR CT compared to the others (13.8 vs 9.5 vs 9.8 mm^3^; *P* = 0.016). No significant differences were observed in the risk profile or echocardiographic characteristics between groups.

**Table 1:** Clinical Characteristics

	Overall (N = 2,025)	T1 (n = 700)	T2 (n = 658)	T3 (n = 667)	*P* Value
Axial angle, °	4.15 ± 2.3	1.88 ± 0.8	3.88 ± 0.5	6.78 ± 1.9	<0.001
Age, y	81.6 ± 6.5	81.3 ± 6.7	81.5 ± 6.4	82.0 ± 6.5	0.10
Women	871 (43.0)	277 (39.6)	282 (42.9)	312 (46.8)	0.027
BMI, kg/m^2^	26.9 ± 5.4	27.2 ± 5.3	27.1 ± 5.4	26.7 ± 5.5	0.15
Diabetes	601 (29.7)	192 (27.4)	197 (29.9)	212 (31.8)	0.20
COPD	180 (8.9)	63 (9.1)	62 (9.5)	55 (8.2)	0.74
STS-PROM score	4.2 ± 3.3	4.0 ± 3.0	4.4 ± 3.8	4.1 ± 3.1	0.077
eGFR, mL/min/1.73 m^2^	55.6 ± 23.7	58.1 ± 24.5	54.5 ± 23.4	54.2 ± 23.7	0.59
AF	632 (31.2)	214 (30.6)	203 (30.9)	215 (32.2)	0.78
Previous PPM	142 (7.0)	50 (7.1)	51 (7.8)	41 (6.1)	0.51
Baseline TTE					
LVEF, %	55.3 ± 13.3	55.2 ± 13.3	55.5 ± 13.3	55.3 ± 13.4	0.93
AVA, cm^2^	0.78 ± 0.2	0.78 ± 0.2	0.77 ± 0.2	0.78 ± 0.3	0.60
Mean gradient, mm Hg	39.8 ± 16.2	39.6 ± 15.2	39.6 ± 16.0	40.0 ± 17.2	0.85
MR ≥ 2	303 (15.0)	103 (14.8)	87 (13.2)	113 (17.0)	0.06
TR ≥ 2	196 (9.7)	74 (10.7)	57 (8.8)	65 (9.8)	0.27
Discharge TTE					
AVA, cm^2^	1.71 ± 0.4	1.71 ± 0.4	1.71 ± 0.4	1.71 ± 0.5	0.95
Mean gradient, mm Hg	10.73 ± 4.4	11.21 ± 4.2	10.80 ± 4.4	10.17 ± 4.6	0.31
Computed tomography					
Aortic annulus perimeter, mm	77.9 ± 8.9	78.3 ± 7.6	76.9 ± 7.9	78.7 ± 3.1	0.20
Aortic angulation, °	50.2 ± 9.5	50.3 ± 9.4	50.2 ± 9.4	50.1 ± 9.7	0.93
Annulus eccentricity	0.76 ± 0.1	0.77 ± 0.1	0.76 ± 0.1	0.76 ± 0.1	0.19
AVC calcium volume, mm^3^	313.6 ± 290.6	307.0 ± 280.3	314.6 ± 276.9	318.8 ± 309.9	0.78
LVOT calcium volume, mm^3^	11.0 ± 2.9	9.8 ± 2.7	9.5 ± 2.3	13.8 ± 3.5	0.016

Nominal data are presented as n (%). Normally distributed continuous data are presented as mean ± SD; skewed distributed data are presented as median ± IQR.

AF = atrial fibrillation; AVA = aortic valve area; AVC = aortic valve calcification; BMI = body mass index; COPD = chronic obstructive pulmonary disease; eGFR = estimated glomerular filtration rate; LVEF = left ventricle ejection fraction; LVOT = left ventricular outflow tract; MR = mitral regurgitation; PPM = permanent pacemaker; STS-PROM = Society of Thoracic Surgeons Predicted Risk of Mortality; TR = tricuspid regurgitation; TTE = transthoracic echocardiography.

### Procedural characteristics

Procedural details are summarized in Table 2. Patients with a bicuspid anatomy represented 4.4% of the population. Technical success was achieved in 91.7% of patients, with no differences between tertiles of coaxiality. Although balloon-expandable valves were the most commonly used devices across all groups, they were less frequently used in the highest tertile compared to the intermediate and the lowest tertiles (58.8% vs 82.1% vs 90.6%, respectively, *P* < 0.001). Patients in the highest tertile received larger valves (mean size 26.7 ± 2.3 mm vs 26.2 ± 2.4 mm vs 26.2 ± 2.3 mm; *P* < 0.001), underwent predilatation less often (37.2% vs 45.9% vs 42.4%; *P* = 0.005), but had postdilatation performed more frequently (24.8% vs 19.3% vs 16.7%; *P* = 0.009), compared to others. There were no significant differences in procedural complications across groups.

**Table 2:** Procedural Characteristics

	Overall (N = 2,025)	T1 (n = 700)	T2 (n = 658)	T3 (n = 667)	*P* Value
Technical success	1,857 (91.7)	639 (91.3)	599 (91.1)	619 (92.8)	0.48
Valve morphology					0.53
Tricuspid	1,958 (95.6)	674 (95.6)	632 (94.9)	652 (96.2)	
Bicuspid	91 (4.4)	31 (4.4)	34 (5.1)	26 (3.8)	
Valve type					<0.001
Balloon-expandable	1,586 (77.4)	639 (90.6)	547 (82.1)	400 (58.9)	
Self-expanding	464 (22.6)	66 (9.4)	119 (17.9)	279 (41.1)	
Valve size, mm	26.4 ± 2.1	26.2 ± 2.2	26.2 ± 2.1	26.7 ± 2.1	<0.001
Oversizing	365 (18.0)	125 (17.9)	128 (19.5)	112 (16.8)	0.44
Undersizing	185 (9.1)	57 (8.1)	57 (8.7)	71 (10.6)	0.24
Predilatation	847 (41.8)	297 (42.4)	302 (45.9)	248 (37.2)	0.005
Postdilatation	409 (20.2)	117 (16.7)	127 (19.3)	165 (24.8)	<0.001
Procedural complications					
Conversion to general anesthesia	63 (3.1)	19 (2.7)	22 (3.3)	22 (3.3)	0.78
Conversion to SAVR	6 (0.3)	1 (0.1)	1 (0.2)	4 (0.6)	0.21
Cardiac tamponade	7 (0.3)	3 (0.4)	2 (0.3)	2 (0.3)	0.89
Hemodynamic instability	22 (1.1)	9 (1.3)	6 (0.9)	7 (1.0)	0.23
MCS unplanned use	5 (0.2)	1 (0.1)	1 (0.2)	3 (0.4)	0.31
Coronary artery occlusion	7 (0.3)	2 (0.3)	1 (0.2)	4 (0.6)	0.36
Annulus rupture/Aortic dissection	20 (1.0)	5 (0.7)	6 (0.9)	9 (1.3)	0.49
Vascular complications	137 (6.7)	52 (7.4)	50 (7.6)	35 (5.2)	0.14

Values are n (%) or mean ± SD.

MCS = mechanical circulatory support system; SAVR = surgical aortic valve replacement.

### 1-month clinical outcomes

**Figure 3:**
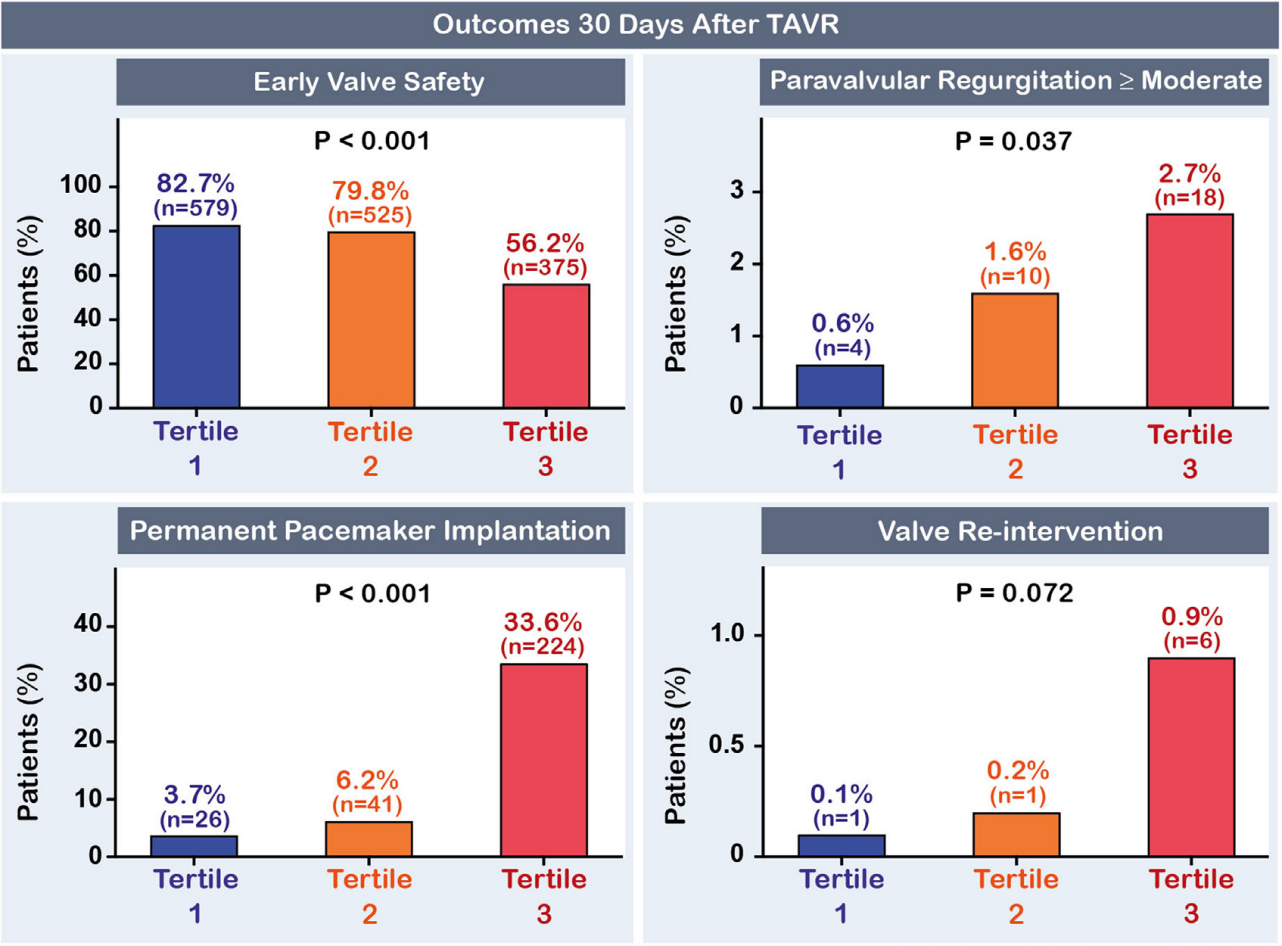
Clinical Outcomes at 30 Days After TAVR Incidence of VARC-3 early safety, moderate or greater paravalvular regurgitation, permanent pacemaker implantation, and reintervention at 30 days after TAVR across tertiles of axial angle. VARC-3 = Valve Academic Research Consortium-3; other abbreviation as in Figure 1.

At the 30-day follow-up, patients in the highest tertile were less likely to achieve device early safety compared to those in the intermediate and lowest tertiles, with rates of 56.2%, 79.8%, and 82.7%, respectively (aHR highest vs lowest tertile 0.67; 95% CI: 0.59-0.76; *P* < 0.001). Overall, 32 patients (1.6%) had at least moderate PVR. Moderate or greater PVR occurred more frequently in patients within the highest tertile compared to those in the intermediate and lowest tertiles, with rates of 2.7%, 1.6%, and 0.6%, respectively (*P* = 0.037). Moreover, 291 patients (14.4%) required a new PPM, 14.0% of those receiving balloon-expandable valves and 15.5% of those receiving self-expanding devices. The incidence of PPM implantation was significantly higher in patients in the highest tertile compared to the other groups with rates of 33.6% vs 6.2% vs 3.7% (aHR highest vs lowest tertile 4.49; 95% CI: 3.65-5.53; *P* < 0.001) (Figure 3). All-cause mortality, stroke, major bleeding, major vascular or cardiac structural complications, and acute kidney disease did not differ across groups.

### One-year clinical outcomes

**Figure 4:**
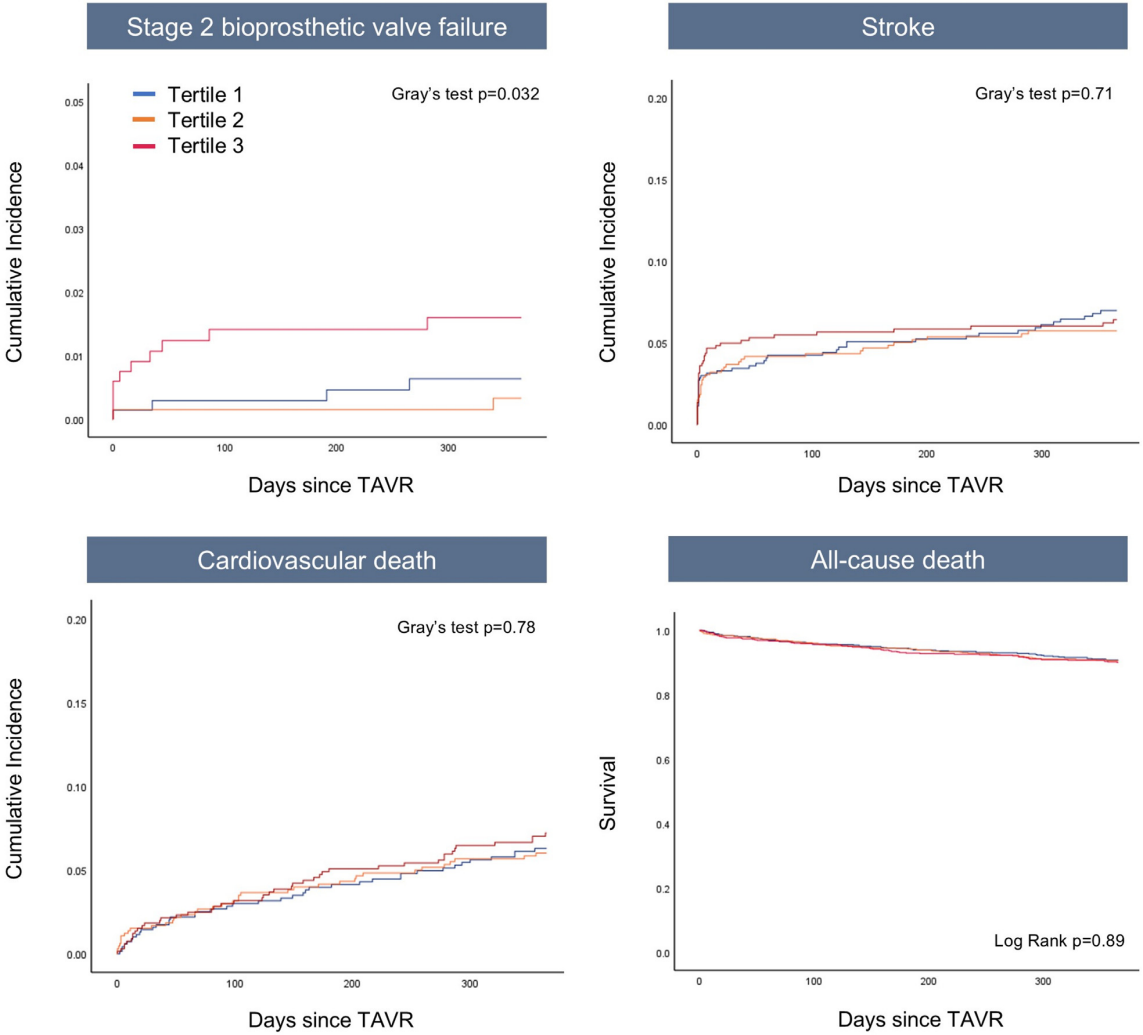
Clinical Outcomes at 1 Year After TAVR Cumulative Incidence of early safety, stroke, cardiovascular mortality, and all-cause death at 1-year post-TAVR across tertiles of axial angle. Abbreviation as in Figure 1.

At 1-year follow-up, device-related reintervention was required in 16 patients (0.8%), with 8 of those adverse events occurring within the first month of the procedure. Eleven patients were treated with surgery, while 5 underwent TAV-in-TAV. The incidence of stage-2 BVF was significantly higher in patients in the highest tertile compared to the intermediate and the lowest tertiles, with rates of 1.5% vs 0.3% vs 0.6%, respectively (HR highest vs lowest tertile 3.47; 95% CI: 1.26-9.54; *P* = 0.016). All-cause mortality, cardiovascular death, and cerebrovascular events rates did not differ between groups (Figure 4).

### Long-term clinical outcomes

At a median follow-up of 370 days (IQR: 245-495 days), 452 patients (22.3%) died. There were no significant differences in clinical outcomes across tertiles of coaxiality, including all-cause mortality, cardiovascular death, cerebrovascular events, and hemodynamic valve deterioration (Supplemental Table 1). While the number of reinterventions was numerically higher in the highest tertile, this difference did not reach statistical significance. Detailed clinical outcomes are presented in Table 3.

**Table 3:** Clinical Outcomes

	T1 (n = 700)	T2 (n = 658)	T3 (n = 667)	HR/aHR^a^ T3 vs T1 95% CI *P* Value
30-d follow-up				
Early safety achieved	579 (82.7)	525 (79.8)	375 (56.2)	0.67 0.59-0.76 <0.001
All-cause mortality	13 (1.9)	13 (2.0)	16 (2.4)	1.24 0.61-2.74 0.57
Stroke	24 (3.4)	24 (3.6)	33 (4.9)	1.26 0.73-2.25 0.41
VARC type 2-4 bleeding	69 (9.9)	74 (11.2)	58 (8.6)	0.90 0.56-1.51 0.71
Major vascular or cardiac complication	69 (9.9)	63 (9.6)	50 (7.5)	0.77 0.55-1.12 0.20
AKI stage 3 or 4	4 (0.6)	9 (1.4)	3 (0.4)	
PVR ≥ moderate	4 (0.6)	10 (1.6)	18 (2.7)	
PPM	26 (3.7)	41 (6.2)	224 (33.6)	4.49 3.65-5.53 <0.001
Reintervention	1 (0.1)	1 (0.2)	6 (0.9)	
1-y follow-up				
Stage-2 BVF	4 (0.6)	2 (0.3)	10 (1.5)	3.47 1.26-9.54 0.016
Longest FU (median 370 d)				
Stage-2 BVF	9 (1.3)	6 (0.9)	12 (1.8)	1.86 0.97-3.58 0.060

Values are n (%).

AKI = acute kidney injury; BEV = balloon-expandable valve; BVF = bioprosthetic valve failure; FU = follow-up; PVR = paravalvular regurgitation; SEV = self-expanding valve; VARC = Valve Academic Research Consortium; other abbreviation as in Table 1.

a Adjusted for valve type (BEV or SEV), age, sex, and STS score.

### Predictors of noncoaxial valve implantation

Higher LVOT calcium volume and the use of self-expanding valves were identified as independent predictors of noncoaxial valve implantation. Conversely, the use of predilatation before TAVR deployment appeared to reduce the likelihood of falling into the highest tertile for axial angle (aOR: 0.79; 95% CI: 0.64-0.98; *P* = 0.036, Supplemental Material).

## Discussion

Achieving optimal bioprosthesis positioning during TAVR can be challenging. Mechanical interactions between the aorta and the prosthetic valve can increase device axial motion along the contralateral side of the landing zone, potentially leading to less accurate valve positioning and negatively affecting acute procedural success. Gorla et al^18^ were the first to describe a “*tilted*” THV configuration resulting from the interaction between the device and the anatomy of the aortic root. In their study, “*tilted*” deployment was defined as an asymmetrical implantation depth between the noncoronary and left coronary cusps, with one cusp implanted at a greater depth than the other.^18^ However, the impact of noncoaxial valve implantation relative to the native annulus plane on clinical outcomes following TAVR has not been previously investigated. In this study, we introduce the concept of valve coaxiality as the angle between a line intersecting the lowest points of the native cusps and the lowest hinge point of the prosthesis frame. This analysis of a large prospective registry cohort of 2,025 patients is the first to examine the clinical outcomes associated with noncoaxial THV implantation, leading to several key findings (Central Illustration): 1) Device early safety is impaired in patients with noncoaxial device deployment; 2) THV axial angle is a strong predictor of stage-2 BVF at 1 year of TAVR; 3) noncoaxial valve implantation affects short-term outcomes, though this effect appears to diminish over time; 4) the degree of LVOT calcifications, along with predilatation and valve type, can increase the risk of noncoaxial THV deployment.

**Central Illustration:**
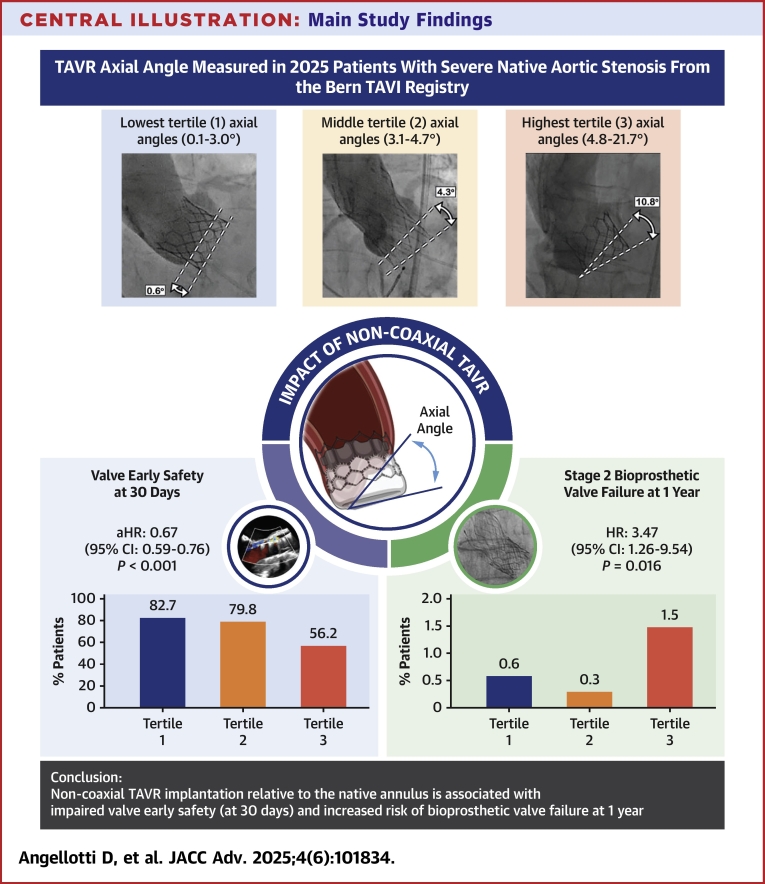
Main Study Findings Noncoaxial transcatheter aortic valve replacement is associated with impaired early outcomes. Abbreviation as in Figure 1.

Despite advances in valve design and implantation techniques leading to a decline in significant PVR following TAVR, rates of moderate or greater PVR at 30 days range from 0.8% to 3.4% with latest-generation devices in low-risk populations.^19^ Moreover, even mild-to-moderate PVR has been associated with an increased risk of mortality at 5 years.^20^^,^^21^ Calcification of the native valve leaflets, commissures, and/or LVOT may lead to noncoaxial THV positioning, which can hinder proper sealing of the device to the surrounding tissue, thereby increasing the risk of PVR. In the present analysis, the risk of moderate or greater PVR was three-fold higher in patients with noncoaxial valve implantation.

Conduction disorders following TAVR remain a significant concern. A meta-analysis of 51,069 patients across 31 observational studies found that PPM implantation after TAVR was associated with an increased long-term risk of all-cause mortality (relative ratio: 1.18; 95% CI: 1.10-1.25; *P* < 0.001).^22^ Mechanical compression leading to temporary inflammation or permanent damage to conduction pathways is likely a primary mechanism in the development of atrioventricular conduction disturbances requiring PPM. In our analysis, the overall incidence of PPM was approximately 1 in 7 patients. Patients with the highest axial angle experienced nearly 4.5 times increased risk of new PPM implantation, suggesting that noncoaxial THV deployment with respect to the annular plane may increase mechanical stress on areas near the conduction system. Acknowledging the growing evidence of their negative impact on long-term prognosis, the VARC-3 composite endpoint of early safety includes both PVR and PPM implantation at 30 days, along with 6 other short-term adverse events.^17^ In this study, nearly 1 in 2 patients with noncoaxial THV implantation experienced compromised early safety, while this proportion decreased to 1 in 5 among patients with coaxial deployment, with this difference primarily driven by PVR and PPM implantation.

With the growing adoption and constant expansion of TAVR to a lower-risk and younger population, the number of patients who outlive their valve and require redo-TAVR is expected to increase considerably. At 10 years follow-up of the NOTION (Nordic Aortic Valve Intervention) trial, the rate of prosthesis reintervention was 4.3%.^23^ In our population, 0.8% of patients underwent reintervention at 1-year follow-up. Patients with noncoaxial valve deployment experienced a more than three-fold increased risk of stage-2 BVF. Of note, 60% of reinterventions occurred within the first month of the procedure. Conversely, at the longest-term follow-up, stage-2 BVF was not significantly impacted by THV coaxiality. Nevertheless, all-cause and cardiovascular mortality did not differ between tertiles at both 1-year and long-term follow-up.

Di Stefano et al^12^ demonstrated that in second-generation valves, the presence of a horizontal aorta had no impact on valve hemodynamics or clinical outcomes. However, patients with an aortic angulation ≥48° exhibited a higher rate of new PPM implantation. In line with these findings, the increased PPM rate in noncoaxial THV implants observed in our cohort supports the idea that more technically demanding procedures, influenced by complex anatomical features such as LVOT calcifications, can affect the performance of the implanted device. Moreover, moderate or severe LVOT calcification has been associated with higher incidence of procedural complications and significant PVR after TAVR.^24^ In this study, LVOT calcium volume appeared to predict noncoaxial valve implantation. THV malpositioning and incomplete tissue sealing may provide a pathophysiological explanation for the link between LVOT calcium presence and the incidence of PVR. On the other hand, balloon predilation of the native valve might help to obtain proper positioning of the THV and consequently a better valve performance. In our study, patients who underwent predilatation had a lower risk of having a noncoaxial device deployment (aOR: 0.79; *P* = 0.036).

Our findings emphasize the importance of coaxial valve implantation. Predilatation may be warranted in patients at high risk of noncoaxial valve deployment. Further studies are needed to assess the risk of premature structural degeneration in patients with suboptimal valve positioning.

### Study Limitations

The primary limitations of this study include its retrospective, single-center design. Coaxiality was evaluated using two-dimensional fluoroscopic images, whereas CT-derived three dimensional imaging could provide a more detailed understanding of the relationship between the native annulus and the device. Our study identified specific independent predictors that may influence the correct apposition of the prosthesis. However, it is important to recognize that these predictors, such as LVOT calcification and the use of self-expanding compared to balloon-expandable THVs, have previously been associated with a higher risk of significant PVR and PPM implantation. Therefore, establishing a causal relationship between noncoaxial THV deployment and these outcomes is not possible through a retrospective analysis. Consequently, this study should be viewed as hypothesis-generating, with the need for further clinical validation through prospective studies. We also did not evaluate the impact of baseline electrocardiogram predictors of conduction disturbances or implantation depth, which could affect the rate of new PPM or influence the interaction between the native annulus and the prosthesis. Despite the availability of data on cardiovascular mortality, the specific cause of death was missing for some patients. For this reason, we were unable to evaluate potential differences in the incidence of stage-3 BVF (device-related death). Finally, given the lack of adjustment for type I error and multiple testing, these results should be interpreted with caution.

## Conclusions

Noncoaxial valve deployment negatively impacts THV early safety and clinical outcomes. An increased axial angle between the prosthesis and the native annulus is associated with a three-fold increased risk of device failure at 1 year after TAVR.

Perspectives **COMPETENCY IN PATIENT CARE AND PROCEDURAL SKILLS:** In a prospective registry of 2,025 patients who underwent TAVR with contemporary devices, patients with noncoaxial TAVR deployment represented a challenging subset, as they faced higher rates of compromised VARC-3 early safety and BVF at short-term follow-up.

**TRANSLATIONAL OUTLOOK:** Suboptimal transcatheter aortic valve positioning may increase the risk of adverse outcomes. Predilatation may be warranted in patients at high risk of noncoaxial valve deployment. Further studies are needed to assess the impact of noncoaxial valve implantation on structural valve deterioration and long-term clinical outcomes.

## Funding support and author disclosures

Dr Angellotti is supported by a research grant provided by the CardioPath PhD program. Dr Maznyczka is an EAPCI (European Association of Percutaneous Coronary Intervention) international structural fellow; her fellowship is funded by 10.13039/100006520Edwards Lifesciences through EAPCI and not directly from Edwards Lifesciences; and has received travel expenses from Edwards Lifesciences, Abbott, Boston Scientific, and Medtronic. Dr Stortecky has received research grants to the institution from 10.13039/100006520Edwards Lifesciences, Medtronic, Abbott, and Boston Scientific; and has received personal fees from Boston Scientific, Teleflex, and BTG. Dr Lanz has received speaker fees to the institution from Edwards Lifesciences and Abbott. Dr Samim has received funding for an online course from Edwards Lifesciences. Dr Reineke has received travel expenses from Abbott, Edwards Lifesciences, and Medtronic and has proctor and consulting contracts with Abbott and Medtronic. Dr Praz has received travel expenses from Edwards Lifesciences, Abbott Vascular, Medira, Siemens Healthineers, and InQB8 Medical Technologies, as well as a research grant to the institution from 10.13039/100011949Abbott Vascular. Dr Windecker has received research and educational grants to the institution from Abbott, Amgen, AstraZeneca, BMS, Bayer, Biotronik, Boston Scientific, Cardinal Health, CardioValve, CSL Behring, Daiichi Sankyo, Edwards Lifesciences, Guerbet, InfraRedx, Johnson & Johnson, Medicure, Medtronic, Novartis, Polares, OrPha Suisse, Pfizer, Regeneron, Sanofi, Sinomed, Terumo, and V-Wave; has served as an unpaid advisory board member and/or unpaid member of the steering/executive group of trials funded by Abbott, Abiomed, Amgen, AstraZeneca, BMS, Boston Scientific, Biotronik, Cardiovalve, Edwards Lifesciences, Med Alliance, Medtronic, Novartis, Polares, Sinomed, V-Wave, and Xeltis but has not received personal payments by pharmaceutical companies or device manufacturers; and has been a member of the steering/executive committee group of several investigator-initiated trials that receive funding by industry without impact on his personal remuneration. Dr Pilgrim has received research, travel, or educational grants to the institution without personal remuneration from Biotronik, Boston Scientific, Edwards Lifesciences, and ATSens; has received speaker fees and consultancy fees to the institution from Biotronik, Boston Scientific, Edwards Lifesciences, Abbott, Medtronic, Biosensors, and Highlife. All other authors have reported that they have no relationships relevant to the contents of this paper to disclose.
