# Supplementary material for: Impact of Noncoaxial Transcatheter Aortic Valve Implantation on Clinical Outcomes
Source: JACC Adv. 2025 May 22;4(6):101834. doi: 10.1016/j.jacadv.2025.101834 (PMC12150034; doi:10.1016/j.jacadv.2025.101834)
Supplement: Supplementary data [file mmc1.docx]

**Supplemental Table 1. Secondary clinical outcomes**

|  | T1  *N*=700 | T2  *N*=658 | T3  *N=667* | HR/aHR*  *95%CI,*  *p-value*  *T3 vs T1* |
| --- | --- | --- | --- | --- |
|  |  |  |  |  |
| **30-days** |  |  |  |  |
| Mean gradient >20 mmHg, *n (%)* | 7  (1.0) | 11 (1.7) | 8  (1.2) |  |
| Device Success  *n (%)* | 599  (85.6) | 563  (85.7) | 577  (86.5) | 0.95  *0.6-2.6*  *0.95* |
| **One-year** |  |  |  |  |
| All-cause Mortality, *n (%)* | 60  (8.6) | 59  (9.0) | 61  (9.1) | 1.16  *0.8-1.7*  *0.43* |
| CV Mortality  *n (%)* | 40  (5.7) | 37  (5.6) | 43  (6.4) | 1.24  *0.8-1.9*  *0.34* |
| CVE, *n (%)* | 45  (6.4) | 36  (5.5) | 41  (6.1) | 0.78  *0.5-1.3*  *0.30* |
| **Longest FU**  ***median 370 days*** |  |  |  |  |
| All-cause Mortality, *n (%)* | 158 (22.6) | 186  (28.3) | 108 (16.2) | 0.93  *0.8-1.4*  *0.37* |
| CV Mortality  *n (%)* | 113 (16.1) | 122 (18.5) | 76 (11.4) | 0.86  *0.7-1.5*  *0.69* |
| HVD ≥ moderate  *n (%)* | 18  (3.4) | 13  (2.5) | 12  (2.6) | 0.94  *0.4-2.0*  *0.86* |
| CVE, *n (%)* | 55  (7.9) | 56 (8.5) | 45 (6.7) | 0.67  *0.4-1.1*  *0.064* |

**Supplemental Table 2.** Predictors of non-coaxial valve implantation.

| **Multivariable logistic regression**  **for highest tertile** | | | | **Multivariable linear regression**  **for axial angle** | | | |
| --- | --- | --- | --- | --- | --- | --- | --- |
|  | OR | *95%CI* | *p-value* | β | *95%CI* | *p-value* |  |
| Age | 1.01 | *0.98-1.10* | *0.477* | 0.004 | *-0.01-0.02* | *0.66* |  |
| Female Sex | 1.21 | *0.92-1.57* | *0.171* | -0.264 | *-0.53-0.06* | *0.055* |  |
| STS-PROM score | 0.98 | *0.94-1.10* | *0.259* | 0.003 | *-0.03-0.33* | *0.86* |  |
| CT-derived  Annulus Area | 1.01 | *0.99-1.04* | *0.205* | 0.001 | *-0.02-0.02* | *0.95* |  |
| CT-derived  Annulus Perimeter | 0.99 | *0.98-1.01* | *0.704* | 0.001 | *-0.05-0.07* | *0.74* |  |
| CT-derived LVOT  calcium volume | 1.04 | *1.02-1.08* | *0.043* | 0.033 | *0.62-2.44* | *0.04* |  |
| Self-Expandable Valve | 5.23 | *3.63-7.52* | *<0.001* | 1.892 | *1.51-2.26* | *<0.001* |  |
| Valve size | 0.99 | *0.91-1.08* | *0.922* | 0.055 | *-0.03-0.14* | *0.22* |  |
| Pre-dilatation | 0.79 | *0.64-0.98* | *0.036* | -0.382 | *-0.63-0.13* | *<0.001* |  |

*CT: computed tomography. LVOT: left ventricle outflow tract.*
